# Antibiotic therapy and outcome from immune-checkpoint inhibitors

**DOI:** 10.1186/s40425-019-0775-x

**Published:** 2019-11-06

**Authors:** David J. Pinato, Daria Gramenitskaya, Daniel M. Altmann, Rosemary J. Boyton, Benjamin H. Mullish, Julian R. Marchesi, Mark Bower

**Affiliations:** 1 Department of Surgery and Cancer, Imperial College London, Faculty of Medicine, Hammersmith Hospital, Du Cane Road, London, W120NN UK; 2 Imperial College London Hammersmith Campus, Du Cane Road, London, W12 0HS UK; 3 Department of Immunology and Inflammation, Faculty of Medicine, Imperial College London, London, W12 0NN UK; 4 Lung Immunology Group, Department of Infectious Disease, Faculty of Medicine, Imperial College London, London, W12 0NN UK; 5 Department of Respiratory Medicine, Royal Brompton & Harefield NHS Foundation Trust, London, SW3 6NP UK; 6 National Centre for HIV Malignancy, Department of Oncology, Chelsea & Westminster Hospital, Fulham Road, London, SW10 9NH UK

**Keywords:** Antibiotics, Immune checkpoint inhibitors, Survival

## Abstract

Sensitivity to immune checkpoint inhibitor (ICPI) therapy is governed by a complex interplay of tumor and host-related determinants. Epidemiological studies have highlighted that exposure to antibiotic therapy influences the probability of response to ICPI and predict for shorter patient survival across malignancies. Whilst a number of studies have reproducibly documented the detrimental effect of broad-spectrum antibiotics, the immune-biologic mechanisms underlying the association with outcome are poorly understood. Perturbation of the gut microbiota, an increasingly well-characterized factor capable of influencing ICPI-mediated immune reconstitution, has been indicated as a putative mechanism to explain the adverse effects attributed to antibiotic exposure in the context of ICPI therapy. Prospective studies are required to validate antibiotic-mediated gut perturbations as a mechanism of ICPI refractoriness and guide the development of strategies to overcome this barrier to an effective delivery of anti-cancer immunotherapy.

## Introduction

Antibiotic therapy has produced unquestionable advances in the management of patients with cancer, a population with intrinsically higher risk of bacterial infection as a result of malignancy or treatment-related immune suppression.

While antimicrobial therapy has markedly reduced morbidity and mortality stemming from infection, the effects of broad-spectrum antibiotics on commensal, non-pathogenic bacterial species have remained for a long time an under-appreciated effect of this therapeutic class of drugs.

The gut microbiota, source of over 100 trillion bacteria, exists in a condition of mutually beneficial relationship with the host. Commensal bacteria are provided with a niche to colonise the host in return for their participation in the digestion of nutrients and xenobiotics, protection from pathogens and shaping of the host’s immune system subsets. Derangement of this delicate relationship has been increasingly well-characterised in the context of tumour-specific immune tolerogenesis [1].

Multiple levels of evidence now support the link between sensitivity to immunotherapy, taxonomic diversity and enrichment in specific gut bacterial taxa, suggesting that some species or species consortia provide intrinsic immune-modulating properties. The landmark study by Gopalakrishnan [2] demonstrated how broader stool bacterial diversity and higher representation of *Ruminococcaceae* communities including *Faecalibacterium* positively influences patients’ survival following ICPI by promoting a strongly immune-reactive microenvironment and lower systemic release of pro-inflammatory cytokines [3]. Many other commensal bacteria have subsequently been recognised to play a similar role including *Bifidobacteria* spp., a saccarolytic Gram-positive genus highly represented within the gut that facilitates dendritic cell maturation and increased accumulation of antigen-specific T-cells within the tumour microenvironment [4]. Similarly, the presence of the anaerobic commensal *Akkermansia Muciniphila* is more common in responders to ICPI, who display higher peripheral CD4 and CD8 memory T-cell responses to this bacterium [5].

Antibiotic (ATB) therapy imposes profound and protracted changes to the taxonomic diversity of the host microbial ecosystem, affecting the composition of up to 30% of the bacterial species in the gut microbiome [6], consequently leading to loss of microbial functions that are protective for the host. Such changes in gut microbial communities are rapid and pervasive, occurring within days from the first antibiotic dose [7] and persisting for up to several months after completion of therapy [8].

Mounting evidence from epidemiological studies has underscored the detrimental role of antibiotics in ICPI outcome, with exposure to antibiotics having been linked to shortened progression-free, overall survival and reduced response rates in patients receiving ICPI as part of clinical trials and in routine practice (Table 1). In a previous study, we demonstrated time-dependence of antibiotics exposure as a strong, tumour-agnostic determinant of outcome in ICPI recipients, confirming prior, but not concurrent antibiotic therapy as doubling the risk of primary progression to immunotherapy and leading to a > 20-months shortening in patients’ survival independent of established prognostic factors and corticosteroid use [10]. Whilst mirroring pre-clinical evidence, where antibiotic pre-conditioning ahead of tumour implantation leads to impaired responses to ICPI in mice [26, 27], the expanding body of clinical studies has so far painted an incomplete picture as to the mechanistic foundations underlying the relationship between ATB and immunotherapy, a point of greater consequence given the potential practice-influencing implications of ATB prescribing in the clinic.

**Table 1:** The relationship between antibiotic exposure and outcomes from immune checkpoint inhibitor therapy

Study	Tumour Sites	ICPI (n, %)	ATB exposure	ATB Duration	ATB Type	Administration route	Response	Survival	Notes
Derosa L et al. [9]	NSCLC (239)	PD-L1 (205, 86%) PD-L1/ CTLA-4 (34, 14%)	pATB (within 30 days) (48, 20%) No ATB (191, 80%)	≤ 7 days (35, 73%) > 7 days (13, 27%)	Beta-lactam (15, 32%) Quinolones (14, 29%) Macrolides (4, 8%) Sulfonamides (12, 25%) Tetracyclines (1, 2%) Nitromidazole (1, 2%) Others (1, 2%)	Oral (42, 87%) IM/ IV (5, 11%) Unreported (1, 2%)	**PD** in 52% exposed vs in 43% unexposed, *P* = 0.26	ATB vs no ATB **median OS**: 7.9 months vs 24.6 months, HR 4.4, 95% CI 2.6–7.7, *P* < 0.01 **median PFS:** 1.9 months vs 3.8 months, HR 1.5, 95% CI 1.0–2.2, *P* = 0.03	Significant impact supported by multivariate analysis
	RCC (121)	PD-L1 (106, 88%) PD-L1/CTLA-4 (10, 8%) PD-L1/Bevacizumab (5, 4%)	pATB (within 30 days) (16, 13%) No ATB (105, 87%)	≤ 7 days (8, 50%) > 7 days (8, 50%)	Beta-lactam (13, 82%) Quinolones (1, 6%) Tetracyclines (1, 6%) Aminoglycosides (1, 6%)	Oral (15, 94%) IV/ IM (1, 6%)	**PD** in 75% exposed vs in 22% unexposed, *P* < 0.01	ATB vs no ATB **median OS:** 17.3 months vs 30.6 months, HR 3.5, 95% CI 1.1–10.8, *P* = 0.03 **median PFS:** 1.9 months vs 7.4 months, HR 3.1, 95% CI 1.4–6.9, *P* < 0.01	
Pinato DJ et al. [10]	NSCLC (119, 60%) Melanoma (38, 20%) Renal (27, 14%) Head & neck (10, 5%) Total *n* = 196	PD-1/PD-L1 (189, 96%)	pATB (29, 15%) (within 30 days) cATB (during ICPI therapy until cessation) (68, 35%) no ATB (99, 50%)	**pATB** ≤7 days (26, 90%) > 7 days (3, 10%) **cATB** ≤7 days (39, 88%)	**pATB** Beta-lactam in 22, 75% **cATB** Beta-lactam in 49, 72%	**–**	**pATB:** **PD** in 80% exposed vs 44% unexposed, *p* < 0.001 **cATB:** **PD** in 50% exposed vs 49% unexposed, *p* = 0.87	pATB (*p* < 0.001) but not cATB (*p* = 0.76) predicted worse OS (26 vs 2 months, HR 7.4, 95% CI 4.2–12.9) Multivariate analysis confirmed pATB as a predictor of OS (HR 3.4, 95%CI 1.9–6.1 *p* < 0.001)	ICPI-refractory in 81% pATB vs 44% no pATB, *p* < 0.001
Hakozaki T et al. [11]	NSCLC (90)	PD-1 (90)	pATB (13, 14%) (30 days before ICPI initiation) no pATB (77, 86%)	≤7 days (1, 8%) > 7 days (12, 92%)	Beta-lactam (8, 61%) Sulfonamides (4, 31%) Quinolones (1, 8%)	Oral (10, 77%) IV (3, 23%)	–	pATB vs no ATB **median PFS:** 1.2 [95% CI, 0.5–5.8] vs 4.4 months [95% CI, 2.5–7.4], *P* = 0.04 **median OS:** 8.8 months vs not reached, *P* = 0.037	Unsupported by multivariate analysis of pATB and OS: HR 2.02, (95% CI, 0.7–5.83, *P* = 0.19)
Galli G et al. [12]	NSCLC (157)	PD-1 (98, 62.4%) PD-L1 (52, 33%) CTLA4 (1, 0.6%) PD-L1/CTLA4 (6, 4%)	ATB: in EIOP (27, 17%) in WIOP (46, 29%) No ATB (111, 71%) High AIER 23 (15%) Low AIER (134, 85%)	Median duration 7.0 days (5.0–33.0)	Quinolone (33, 72%) Macrolide (8, 17%) Beta-lactam (14, 30%) Rifaximin (4, 8.7%)	Oral (44, 98%) IM (3, 6.5%), IV (2, 4.4%).	Exposed in EIOP **RR**: 11.1% vs 24.6%, *p* = 0.20; **DCR**: 51.9% vs 56.2%, *p* = 0.8319. **AIER (high vs low)** **RR**: 8.7%, vs 26.6%. *p* = 0.11 **DCR**: 47.8% vs 56.0%, *p* = 0.50,	High vs low AIER **median PFS:** 1.9 [95% CI, 1.3–3.0] vs 3.5 months [95% CI, 2.6–5.0] *p* < 0.0001 **median OS:** 5.1 [95% CI, 3.8–5.9] vs 13.2 months [95% CI, 9.9–5.9] *p* = 0.0004	Exposed vs unexposed in EIOP **median PFS:** 2.2 [95% CI, 1.8–3.2] vs 3.3 months [95% CI, 2.6–4.8] *P* = 0.1772 **median OS:** 11.9 [95% CI, 9.2–15.6] vs 5.9 months [95% CI, 4.5–22.5] *P* = 0.2492 Significant impact supported by multivariate analysis
Ahmed J et al. [13]	NSCLC (34, 57%) Renal (4, 7%) HCC (5, 8%) Urothelial (5, 8%) Other (12 20%) Total *n* = 60	ICPI with chemotherapy (8, 13%) PD-1 (49, 82%) PD-L1 (3, 5%)	pATB or cATB (2 weeks before or after ICPI initiation) (17, 28%) No ATB (43, 72%)	8–14 days	Beta-lactam (14, 82%) Quinolone (5, 29%) Vancomycin (7, 41%) Daptomycin (1, 6%) Linezolid (2, 12%) Meropenem (3, 18%) Tetracyclines (2, 12%) Bactrim (1, 6%) Azithromycin (1, 6%) Nitrofurantoin (1, 6%)	**–**	**RR**: 29.4% in exposed vs 62.8% in unexposed, *p* = 0.024	Decreased **PFS** with ATB HR 1.6; 95% CI: 0.84–3.03, *p* = 0.048 **Median OS:** 24 in exposed vs 89 months in unexposed *p* = 0.003	Narrow-spectrum ATB alone did not affect the RR, but broad-spectrum ATB decreased RR (*p* = 0.02) and PFS (*p* = 0.012). Multivariate analysis found that only ATB decreased RR (*p* = 0.0038) and PFS (*p* = 0.01)
Tinsley N et al. [14]	Melanoma (206, 66%) NSCLC (56, 18%) Renal (46, 15%) Total *n* = 303	–	pATB or cATB (2 weeks before or 6 weeks after ICPI initiation) (94,31%)	–	The commonest ATBs: beta-lactam and macrolides	–	–	ATB vs no ATB **PFS** 97 (95% CI 84–122) vs 178 days (95% CI 155–304) *p* = 0.049 **OS** 317 days (95% CI 221–584) vs 651 days (95% CI 477–998) *p* = 0.001.	Cumulative ATB (> 10 days, multiple concurrent/successive courses) further shortened PFS to 87 days (95% CI 83–122) *p* = 0.0093 and OS to 193 days (95% CI 96–355) *p* = 0.00021 pATB exposed had shorter PFS and OS than cATB exposed (HR 1.37, *p* = 0.29 and HR 1.72, *p* = 0.08)
Khan U et al. [15]	Lung (111, 46%) Bladder (36, 15%) Renal (35, 14%) GI (16, 7%) Other (44, 18%) Total *n* = 242	PD-1 (189, 78%) PD-L1 (52, 21%)	75, 46 and 32% received ATBs within 6 months, 60 days and 30 days of starting ICPIs	–	–	–	cATB use in the first 30- or 60-days of ICPI therapy associated with inferior **ORR** (OR 0.40, *p* = 0.01 and OR 0.42, *p* = 0.005, respectively)	**–**	pATB or cATB use in the first 6 months of ICPI use had no impact
Routy B et al. [5]	NSCLC (140, 56%), RCC (67, 27%) urothelial carcinoma (42, 17%) Total *n* = 249	PD-1/PD-L1 (249, 100%)	pATB or cATB (2 months before or 1 month after ICPI initiation) (69, 28%) no ATB (180, 72%)	–	β-lactam+/− inhibitors, fluoroquinolonesor macrolides	Mostly oral	–	ATB vs no ATB For all groups combined **median PFS:** 3.5 vs 4.1 months *p* = 0.017 **median OS:** 11.5 vs 20.6 months *p* < 0.001 For individual cancer groups, PFS and/or OS were also shorter in ATB group	Univariate and multivariate Cox regression analyses confirmed the negative impact of ATB, independent from other factors
Mielgo-Rubio X et al. [16]	NSCLC (168)	PD-1 (168,100%)	pATB or cATB (2 months before or 1 month after ICPI initiation) (47.9%) No ATB (52.1%)	–	–	Oral (70%) IV (30%)	–	ATB vs no ATB **OS:** 8.1 (95%CI 3.6–12.5) vs 11.9 months (95%CI 9.1–14.7) *p* = 0.026 **PFS:** 5 (95%CI 3.1–6.9) vs 7.3 months (95%CI 2–12) *p* = 0.028	IV ATB had a more negative impact than oral ATB **OS:** 2.9 (95%CI, 1.6–4.1) vs 14.2 months (95%CI, 7.9–20.6) *p* = 0.0001 **PFS:** 2.2 (95%CI 0.6–3.7) vs 5.9 months (95%CI 3.9–8) *p* = 0.001
Ouaknine J et al. [17]	NSCLC (72)	PD-1 (72,100%)	pATB or cATB (2 months before or 1 month after ICPI initiation) (30, 42%) No ATB (42, 58%)	Median duration 9.5 days (IQR 7–14)	The commonest ATBs: β-lactam and vancomycin	Mostly oral (65%)	ATB vs no pATB **ORR** 37% vs 24% *p* = 0.276 **Clinical benefit rate** 27% vs 29% *p* = 0.859	ATB vs no ATB **median OS:** 5.1 (IQR 3.4-not reached) vs 13.4 months (IQR 10.6-not reached) *p* = 0.03 **median PFS:** 2.8 (IQR 1.4–5.1) vs 3.3 months (IQR 1.8–7.3) *p* = 0.249	–
Kaderbhai C et al. [18]	NSCLC (74)	PD-1 (74, 100%)	pATB (within 3 months) (15, 20%) No ATB (59, 80%)	–	–	–	No difference in ORR *p* = 0.75	No difference in **PFS** and *p* = 0.72,	–
Zhao S et al. [19]	NSCLC (109)	PD-1 (57, 52%) PD-1/ chemotherapy (33, 30%) PD-1/apatinib or bevacizumab (19, 18%)	pATB or cATB (1 month before or after ICPI initiation) (20, 18%) No ATB (89, 82%)	–	The commonest ATBs: β-lactam inhibitors and fluoroquinolones	–	Higher **PD** rates in ATB-treated group (*p* = 0.092)	ATB decreased **PFS**, *p* < 0.0001 and **OS**, *p* = 0.0021	In multivariable analysis, ATB was associated with shorter PFS (HR = 0.29, 95%CI 0.15–0.56, *p* < 0.0001) and OS (HR = 0.35, 95%CI 0.16–0.77, *p* = 0.009)
Thompson et al. [20]	NSCLC (74)	PD-1 (74, 100%)	pATB (within 6 weeks) (18, 24%) No ATB (56, 76%)	–	Mostly fluoroquinolones (50%)	–	ORR in ATB vs no ATB groups 25% vs 23% (adjusted OR 1.2, *p* = 0.20).	ATB vs no ATB **PFS** 2.0 vs 3.8 months *p* < 0.001) **OS** 4.0 vs 12.6 months, *p* = 0.005	The impact of ATB on PFS and OS was independent of other factors (HR 2.5, *p* = 0.02), (HR 3.5, *p* = 0.004), respectively
Derosa L et al. [21]	RCC (80)	PD1/PD-L1 (67, 84%), PD-1/CTLA-4 (10, 12%) PD-L1/ bevacizumab (3, 4%)	pATB (within 1 month) (16, 20%) No ATB (64, 80%)	–	Mostly β-lactam and fluoroquinolones	–	Lower **ORR** in ATB group vs no ATB *p* < 0.002	ATB vs no ATB **PFS** 2.3 vs. 8.1 months, *p* < 0.001	Confirmed by multivariate analysis
Do TP et al. [22]	Lung (109)	PD-1 (109, 100%)	pATB or cATB (1 month before ICPI or concurrently) (87, 80%) No ATB (22, 20%)	–	β-lactam (12, 13.8%) quinolones (11,12.6%) other (7, 8.1%) multiple antibiotics (57, 65.5%)	–	–	ATB vs no ATB **OS** 5.4 vs 17.2 months (HR 0.29, 95% CI 0.15–0.58 *p* = 0.0004)	
Elkrief A et al. [23]	Melanoma (74)	PD-1 (54, 73%) CTLA-4 (5, 6.8%) CTLA-4/ carboplatin/paclitaxel (15, 20%)	pATB (within 1 month) (10, 13.5%) No ATB (64, 86.5%)	> 7 days (7, 70%) < 7 days (3, 30%)	Mostly β-lactams± inhibitors	Oral (40%) IV (60%)	**ORR** ATB vs no ATB 0% vs 34%	ATB vs no ATB **median PFS** 2.4 vs 7.3 months (HR 0.28, 95% CI 0.10–0.76 *p* = 0.01) **median OS** 10.7 vs 18.3 months (HR: 0.52, 95% CI 0.21–1.32 *p* = 0.17).	The multivariate analysis supported the impact of ATB on PFS (HR 0.32 (0.13–0.83) 95% CI, *p* = 0.02).
Huemer F et al. [24]	NSCLC (30)	PD-1 (30, 100%)	pATB or cATB (1 month before or 1 month after ICPI initiation) (11, 37%) No ATB (19, 63%)	–	β-lactam (7, 64%), fluoroquinolones (4, 36%) and carbapenems (2, 18%)	–	–	ATB vs no ATB **median PFS** 3.1 vs 2.9 months, (HR = 0.46 95%CI: 0.12–0.90 *p* = 0.031). **median OS** 15.1 vs 7.5 months (HR = 0.31 95%CI: 0.02–0.78 *p* = 0.026).	The multivariate analysis supported the impact of ATB on PFS (*p* = 0.028) and OS (*p* = 0.026).
Lalani A et al. [25]	RCC (146)	PD-1/PD-L1 (146, 100%)	pATB or cATB (2 months before or 1 month after ICPI initiation) (31, 21%) No ATB (115, 79%)	–	–	–	ATB vs no ATB **ORR** 12.9 vs 34.8% *p* = 0.026	ATB vs no ATB 2.6 (1.7–5.3) vs 8.1 (5.6–10.9) months *p* = 0.008	–

Abbreviations: *EIOP* (Early Immunotherapy Period): antibiotics given between 1 month before and 3 months after starting immunotherapy, *WIOP* (Whole immunotherapy Period): antibiotics given throughout immunotherapy, cumulative exposure to antibiotics; AIER defined as “days of antibiotic therapy/days of immunotherapy’: AIER stratified over the median (4.2%) into high and low AIER groups, *RR* Response rate, *DCR* Disease control rate, *GI* Gastrointestinal, *ORR* Overall response rate, *IV* Intravenous, *IM* Intramuscular

Most of the studies highlighting the importance of a healthy gut microbial environment as a pre-requisite for ICPI response were unfortunately characterised by insufficient data on preceding or concomitant antibiotic exposure, making it impossible to disentangle the role of antibiotic-induced perturbation of the gut ecosystem in influencing clinically meaningful outcomes in these patients [3].

Mechanistically, the breadth and depth of downstream effects produced by antibiotics within the cancer-immune synapse are an important challenge in studying this prognostically adverse relationship. On one hand, the direct bacteriostatic/bactericidal effect of antibiotics can cause selective pressure within the host microbial ecosystem and instigate an alternative microbiota state characterised, amongst other traits, by downregulation of major histocompatibility complex (MHC) class I/II genes and impaired effector T-cell responses, immunologic traits implicated in reduced responsiveness to ICPI [28].

ATB-induced depletion of gut bacteria can also shift the repertoire of microbial-associated molecular patterns (MAMPS). These molecules signal through mucosal innate immune cells primarily via toll-like receptors (TLRs) and NOD1 [29] to influence neutrophil priming, reduce local cytokine release and prime adaptive immunity by influencing the expression of MHC genes within the intestinal mucosa and reduce immunoglobulin secretion [30]. Antibiotic treatment impairs TH_1_/TH_17_ responses in tumour-bearing mice through direct pre-conditioning of the gut microbiota, reducing the efficacy of cyclophosphamide-mediate immune-rejection of the tumour [31]. In addition, antibiotics can also reduce the capacity of adoptively transferred CD8+ T-cells to mediate a tumour-specific response through altered LPS/TLR4 signaling in lymphodepleted mice [32].

By disrupting the gut ecosystem, antibiotics instigate downstream metabolic alterations within the microenvironment with complex repercussions to the tumour-host-microbe interface. Amongst them, changes in the availability of short-chain fatty acids produced by *Akkermansia, Faecalibacteria* and *Enterococcus* from the catabolism of non-digestible carbohydrates and the conversion of primary bile acids to secondary bile acids (including deoxycholate) mediated by *Clostridiales* can significantly alter gut homeostasis and lead to profound and clinically meaningful immune-modulatory consequences [33]. The immune-metabolic repercussions secondary to gut dysbiosis, potentially reversible by oral *Akkermansia* supplementation [34], might explain the influence of body mass index in determining response to ICPI [35, 36].

With improved characterization of immune-microbiologic underpinning of the relationship between antibiotics and ICPI outcome, a key question now is whether disruption of a well-equilibrated gut bacterial ecosystem is truly causal in this relationship, and thus whether reversal of antibiotic-mediated gut dysbiosis might prove beneficial in restoring full sensitivity to ICPI. Whether a favourable gut microbiota is a reflection of an otherwise healthy host rather than the *primum movens* of clinically meaningful anti-cancer immune responses is still the subject of intense debate [13]. To this end, appreciating how antibiotics might dynamically affect such a strong immune-microbiologic correlate of response to checkpoint inhibition is of key importance to pave the way for strategies that could restore or protect the integrity of this important phenotypic correlate of response. To address the multiplicity of mechanisms that are likely to underscore this complex and bi-directional relationship, the coordinated study of a number of fundamental pathophysiologic processes including bacterial translocation, immune-modulation, an altered metabolome, enzymatic degradation and reduced diversity of the gut microbiome has been proposed as an overarching framework [37].

Gaining sufficient insight as to the mode of action by which bacteria might work as biotherapeutic agents is not just important for patient prognostication, but is in fact key to a successful, rational development of microbiome-modulating therapies which improve patient’s outcome with ICPI. With antibiotic use now having been validated as an important and dynamic factor influencing outcome from immunotherapy, concerted efforts should be aimed at characterizing the candidate taxonomic features in the gut microbiota that are associated with worse outcome from ICPI in the context of preceding and concomitant antibiotic exposure and evaluate them in conjunction with the concomitant prescription of proton pump inhibitors, corticosteroids and vaccines, all of which have been postulated to influence ICPI response [38].

Recognising these changes is expected to facilitate the clinical development of diverse biotherapeutic approaches to induce microbiome reprogramming including dietary interventions with pre-biotics, therapeutic administration of single or multiple types of bacterial species or their metabolites, selective antibiotic therapy or faecal microbial transplantation, all of which are currently at the focus of intense clinical research efforts [26].

## Data Availability

n/a.

## Abbreviations

ATB: Antibiotic
cATB: Concurrent antibiotic treatment
CD: Cluster of Differentiation
CTLA-4: Cyotoxic T-Lymphocyte Associated Protein 4
DCR: Disease control rate
EIOP: Early Immunotherapy Period
GI: Gastrointestinal
HR: Hazard Ratio
ICPI: Immune-checkpoint inhibitors
LPS: Lipopolysaccharide
MHC: Major Histocompatibility Complex
NOD1: Nucleotide-binding oligomerization domain-containing protein 1
NSCLC: Non-small Cell Lung Cancer
ORR: Overall response rate
OS: Overall Survival
pATB: Prior antibiotic treatment
PD: Progressive Disease
PD-1: Programmed Cell-Death 1
PD-L1: Programmed Cell-Death Ligand 1
PFS: Progression-free survival
RCC: Renal Cell Carcinoma
RR: Response rate
TH: T-Helper cell
TLR: Toll-like receptors
WIOP: Whole immunotherapy Period
